# Spatial-Temporal Dynamics of Cropping Frequency in Hubei Province over 2001–2015

**DOI:** 10.3390/s17112622

**Published:** 2017-11-14

**Authors:** Jianbin Tao, Wenbin Wu, Wenbin Liu

**Affiliations:** 1 Key Laboratory for Geographical Process Analysis & Simulation of Hubei Province/College of Urban and Environmental Sciences, Central China Normal University, Wuhan 430079, China; taojb@mail.ccnu.edu.cn (J.T.); liuwenbin_ccnu@163.com (W.L.); 2 Key Laboratory of Agricultural Remote Sensing, Ministry of Agriculture/Institute of Agricultural Resources and Regional Planning, Chinese Academy of Agricultural Sciences, Beijing 100081, China

**Keywords:** spatial-temporal dynamics, cropping frequency, phenology, time-series MODIS data, hierarchical clustering method

## Abstract

Mapping crop patterns with remote sensing data is of great importance for agricultural production, food security and agricultural sustainability. In this paper, a hierarchical clustering method was proposed to map cropping frequency from time-series Moderate Resolution Imaging Spectroradiometer (MODIS) Enhanced Vegetation Indices (EVI) data and the spatial and temporal patterns of cropping frequency from 2001 to 2015 in Hubei Province of China were analyzed. The results are as follows: (1) The total double crop areas decreased slightly, while total single crop areas decreased significantly during 2001 and 2015; (2) The transfer between double crop and single crop was frequent in Hubei with about 11~15% croplands changed their cropping frequency every 5 years; (3) The crop system has obvious regional differentiation for their change trend at the county level.

## 1. Introduction

Food security is increasingly becoming a research focus in international community due to increasing population, rapid urbanization and global climate change [1,2]. Crop mapping is a key issue in this context. It’s of great value to obtain accurate crops information on regional or national scale timely and to update it regularly using remote sensing images. Information about the extent and intensity of multiple cropping is very important to ensure food security and to understand the carbon exchange processes in agro-ecosystems [3,4,5]. The spatial patterns of crops as well as their temporal and spatial dynamics lay the foundation for analysis of the mechanisms of dynamic in crop spatial patterns, the establishment of simulation models, researches on the contributions of agricultural ecosystems to the terrestrial carbon cycle and evaluation of the influence of global changes on regional agricultural productions [6]. Therefore, monitoring the changes of spatial patterns of crop is of great significance for food security and development of agriculture.

Time-series vegetation indices have been widely used in the remote sensing community. The results of previous studies showed that vegetation indices with moderate spatial resolution (from hundreds of meters to one kilometer) and high temporal frequency (revisited every few days or even daily), obtained from Moderate Resolution Imaging Spectroradiometer (MODIS), Advanced Very High Resolution Radiometer (AVHRR) and SPOT, could be used to identify crop growth curves and multiple cropping areas on a large scale. Moderate spatial resolution time-series vegetation indices have successful applications on crop mapping in Brazil [7,8,9,10], Thailand [11], Vietnam [12,13], Italy [14], Canada [15], USA [16,17], Laurentian Great Lakes Basin [18], Central Asia [19], Africa [20,21], Europe [22] and China [23,24,25,26]. Lunetta et al. [18] examined the crop rotational patterns across the Great Lakes Basin using map products derived from MODIS-NDVI (Normalized Difference Vegetation Index) data. Ding et al. [25] extracted and analyzed the spatial and temporal changes of multiple cropping index from 1999 to 2013 in China using 10-day SPOT time-series (NDVI).

MODIS data has the advantages of frequent coverage and moderate spatial resolution and is suitable for mapping cropping frequency at regional and national scales. Most previous MODIS-NDVI crop mapping applications have focused on single year crop mapping [18] or single crop system. The crop patterns have been intensively investigated in Northeast or North China in previous studies, however there has been few studies in Central and South China. China has a vast territory width with significantly varied natural environments. Crop rotation, double crop or even triple crop are very common in China. The phenomena of mixed pixels are more often observed on MODIS data in Central and South China due to the fragmented cropland fields and at the same time constrained by local natural environment and farmer’s planting behavior. Therefore, mapping crop patterns in Central and South China is more challenging concerning classification method and has great research value in remote sensing of agriculture.

Time-series NDVI data from MODIS satellite sensor carry useful information about the seasonal vegetation development and this information will facilitate the analyses of spatial and temporal characteristics of the crop pattern. Phenology is the response of the vegetation to seasonal climatic cycles in irradiance, temperature and rainfall. Crops have quite a different phenological calendar from natural vegetation, showing distinct phenological characteristics differing from those of the background land-cover types.

The objective of this study is to explore the spatial and temporal patterns of the crop system using time-series MODIS data in Hubei Province. The specific objectives include: (1) Developing a hierarchical clustering method (HCM) to map cropping frequency based on time-series MODIS data; (2) Introducing statistics method to measure the change trend of cropping frequency, including change direction and their significance level; (3) Analysis of the spatial-temporal patterns of cropping frequency, obtaining the knowledge of the crop system in Hubei Province, providing a basis for agriculture management. The novelty of the research is that, a HCM with two-stage unsupervised classification was proposed to map the cropping frequency. Key phenological parameters, including the start of season (SOS), the end of season (EOS) and the length of season (LOS), were extracted using dynamic threshold methods. Cropping frequency maps for 2001–2015 were obtained combining time-series Enhanced Vegetation index (EVI) data and vegetation phenological data. The spatial and temporal dynamics over 15 years were analyzed using statistical methods and the reasons for the change of crop patterns in Hubei Province were also discussed.

This paper is organized as follows. Section 2 introduces the research area and the experimental materials. Section 3 introduces the method of extracting vegetation phenological parameters and the hierarchical clustering method for mapping cropping frequency. In Section 4, the spatial-temporal dynamics of the cropping frequency are analyzed using statistical methods. In Section 5, the reasons of the change trend are discussed. Finally, conclusions are stated in Section 6.

## 2. Research Area and Data

### 2.1. The Research Area

**Figure 1:**
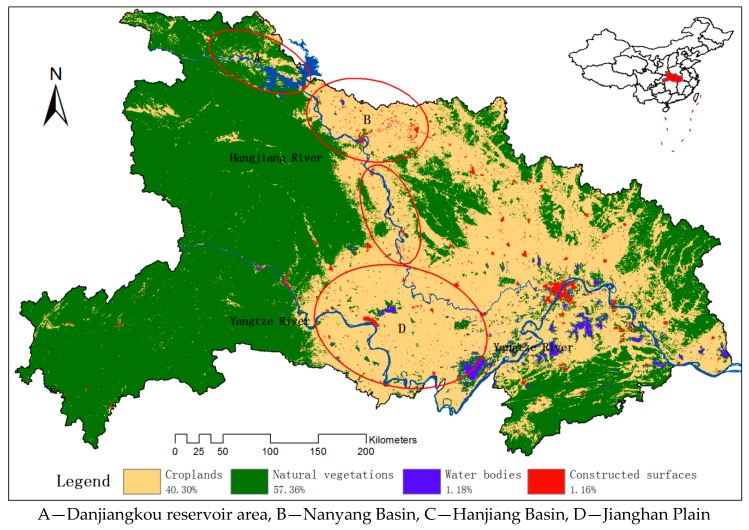
The research area and the main land-cover classes in 2010 from MCD12Q1 UMD datasets, which was from the standard Moderate Resolution Imaging Spectroradiometer (MODIS) land cover type data product in the IGBP Land Cover Type Classification.

The Hubei Province of China was chosen as the research area (Figure 1). Hubei is located in Central China. It has a humid subtropical climate with four distinct seasons. The average temperatures are 1 to 6 °C in January and 24 to 30 °C in July. The annual rainfall is between 800–1600 mm on average. The two major rivers of Hubei are the Yangtze River and its largest branch, the Hanjiang River. Croplands take up 40.30% of the total land resources. The Jianghan Plain is a main grains productive basement of China, which takes up most of central and southern Hubei, while the west and the peripheries are more mountainous. The dominant land-cover classes in the research area are forests, shrublands, grasslands, croplands, water bodies and constructed surfaces. Forests, shrublands, grasslands are combined to a more general type, natural vegetation, since we focus on croplands and will not classify the natural vegetation precisely. As for croplands, single crop and double crop coexist in Hubei and single crop is dominant is this area.

### 2.2. The Experimental Data

The data used in this study were the MOD13Q1 v006 data, consisting of MODIS products generated from data by EOS/Terra Satellite covering years 2001 to 2015. The products include 250 m resolution NDVI and EVI data, reflectance data and quality control data, which were synthesized over 16 days based on the Maximum Value Composite (MVC) method. The products were corrected geometrically and atmospherically. The dataset was downloaded from the website of the U.S. NASA LP DAAC working group (https://lpdaac.usgs.gov/lpdaac/products/modis_products_table). The MODIS image sequence numbers of the tile covering the research area is h27v05, h2706, h2805, h2806 (h: horizontal, v: vertical). In addition, a data pixel reliability layer was extracted from the MOD13Q1 v006 products, the spatial and temporal resolutions of which were consistent with the NDVI and EVI datasets. Since NDVI is chlorophyll sensitive and the EVI is more responsive to canopy structural variations, including leaf area index (LAI), canopy type, plant physiognomy and canopy architecture [27], EVI data layer was extracted as the vegetation indices datasets in this research. The MCD12Q1 land-cover data, Landsat image, along with Google Earth high-resolution imagery were analyzed together to interpret and validate the cropping frequency map visually.

Geographical geometric correction, image clipping and re-sampling were performed on the dataset using MODIS Reprojection Tool (MRT). The method adopted for sampling was nearest neighbor. All the datasets were re-projected into geographic coordinates of latitude and longitude on the WGS 1984 coordinate reference system.

## 3. Methods

The proposed method for mapping cropping frequency consists of three main components: (1) extraction of the vegetation phenological parameters based on time-series MODIS data using TimeSat; (2) a hierarchical clustering method for cropping frequency mapping using phenological parameters and (3) temporal-spatial analysis of the crop patterns using spatial analysis and statistics test.

### 3.1. Phenological Parameters Extraction

Phenological parameters were extracted using the TimeSat software package [28]. The Savitzky-Golay (S-G) filtering method was used in this research to smooth the time-series MODIS data, fitting the variation tendency of time-series EVI data with an envelope curve. The fitting was optimized in an iterative process and a smoothly curve describing the time-series EVI data could therefore be reconstructed. The above process reduced the noise effects induced by clouds and also clearly revealed the phenological pattern contained in the temporal vegetation index profile. The Logistic model was then utilized to extract critical seasonality parameters.

**Figure 2:**
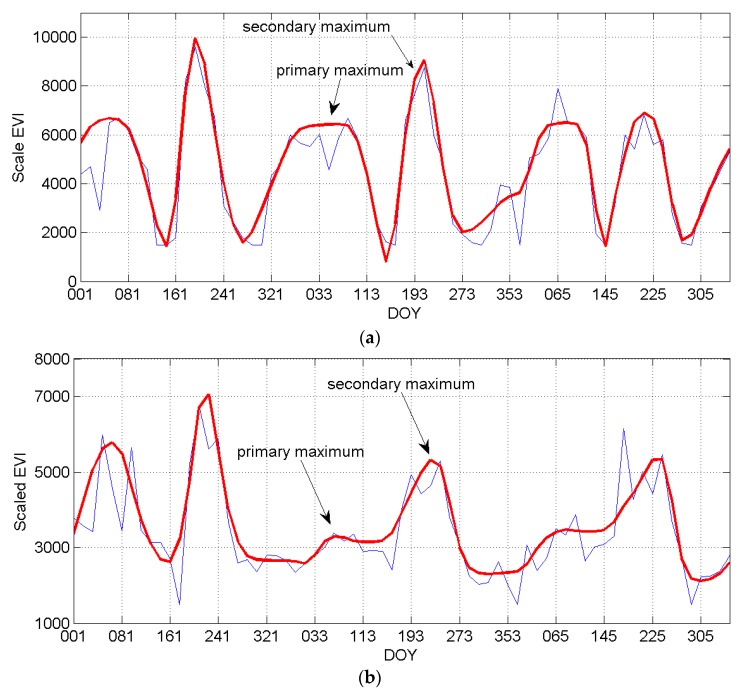
Profiles of time-series (EVI) data, (**a**) for two annual seasons and (**b**) for one annual season. DOY, the day of a year.

Dynamic threshold was used to determine the number of seasons. In TimeSat, the fitting procedure always gives a primary maximum and a secondary maximum. The primary maximum is usually smaller than the secondary maximum in the research area, differing from those in the reference paper [29]. If the amplitude ratio between the primary maximum and the secondary maximum exceeds a user defined threshold, the curve was considered as having two annual seasons (Figure 2a). Otherwise, the curve has only one annual season Figure 2b [29]. In this research, the seasonality parameter was set to 0.3 to carefully discriminate between noise and a second annual season. Once the number of seasons was determined, all the parameters need can be computed for each of the full seasons in the time-series.

**Figure 3:**
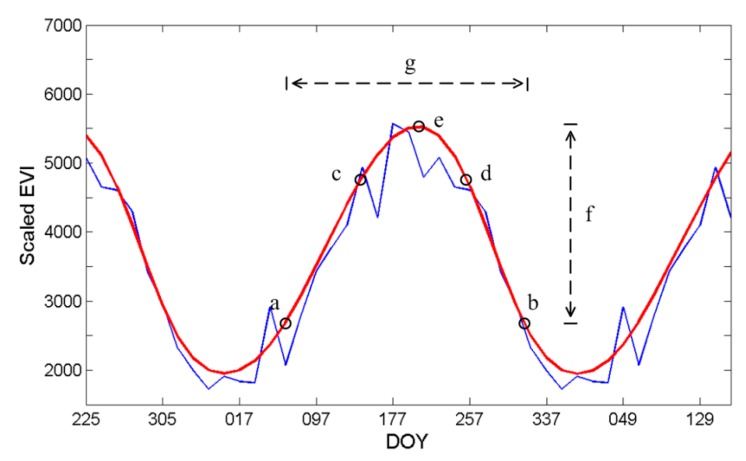
Points (**a**,**b**) mark, respectively, start and end of the season. Points (**c**,**d**) give the 80% levels. (**e**) displays the point with the largest value. (**f**) displays the seasonal amplitude and (**g**) the seasonal length. The blue line indicates the time-series EVI data and read line indicates the fitting curve of the original data.

In the current version of TimeSat a number of key seasonality parameters such as the time of the start and end of the season, the largest value, the length of the season and the amplitude are computed for each of the full seasons in the time-series [29]. Some of these parameters are displayed in Figure 3.

In our research, start of season (SOS), end of season (EOS), length of season (LOS), was extracted for the first season. The extracted parameters were then employed together with the land-cover maps to facilitate the cropping frequency mapping for the research area.

### 3.2. Hierarchical Clustering Method

A hierarchical clustering method (HCM) was proposed to map the cropping frequency. In this method, clustering was conducted in two successive stages, in which different feature space was constructed.

**Figure 4:**
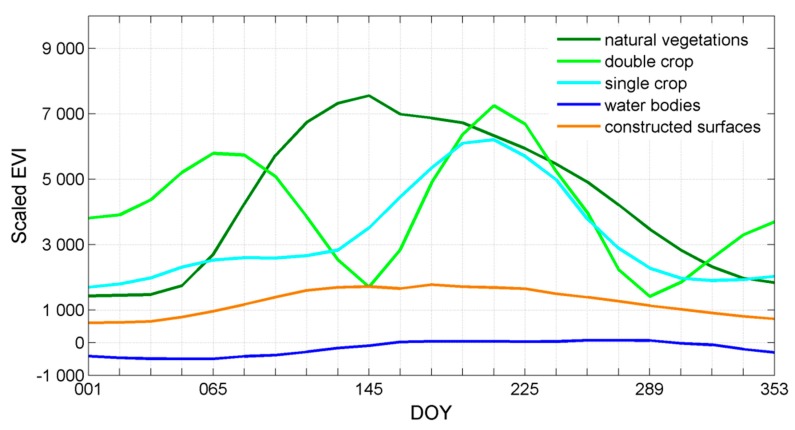
EVI curves for main land-cover classes in Hubei Province. DOY, the day of a year. EVI, enhanced vegetation index.

Time-series EVI data carry useful phenological information for different land-cover classes. Time-series EVI sample data from research area was plot in Figure 4, in which the distinct pattern among natural vegetation, double crop, single crop, water bodies and constructed surfaces can be observed.

**Figure 5:**
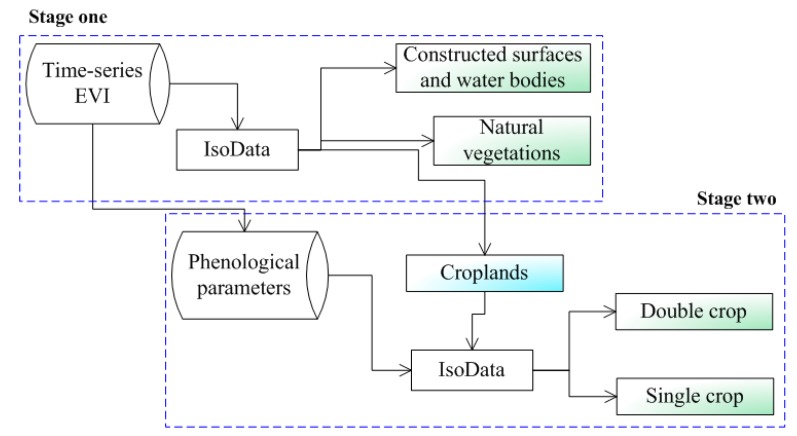
Flowchart of hierarchical clustering method.

In stage one of HCM, a feature space including 23 layers of time-series EVI data was constructed to distinguish the main land-cover classes according their unique phenological calendar. The EVI curve for water bodies was low and close to zero. The EVI curve for constructed surfaces was relatively low and no obvious trend can be observed in its curve, indicating a lack of apparent pattern in their time-series EVI values. However, the EVI curves for natural vegetation, double crop and single crop exhibit a significant peak around DOY (Day of a Year) 145, 065 and 225 respectively and also with a higher EVI value. Among all the land-cover classes, only the double crop has double peaks within about five months (growing season in summer), indicating its growth decreased rapidly and then recovered rapidly when the second season started. Also, with this unique feature space we classify the image into three more general categories—croplands, natural vegetation, constructed surfaces and water bodies using IsoData with the 23 layers of EVI data (Figure 5). Then, natural vegetation, water bodies and constructed surfaces were masked out. Subsequently, our research will focus on croplands in stage two of HCM.

**Figure 6:**
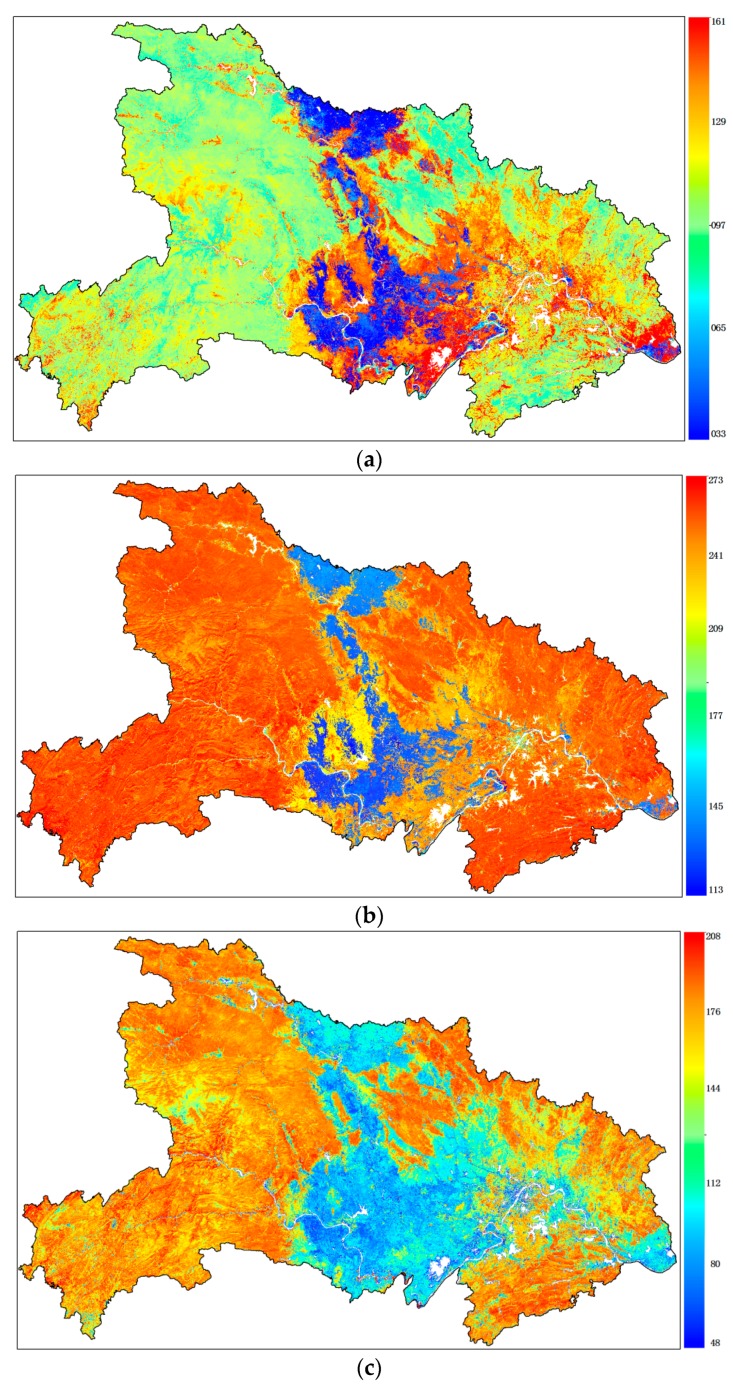
Three phenological parameters, (**a**) start of season (SOS), (**b**) end of season (EOS) and (**c**) length of season (LOS) of the first season in 2015. Color bars in (**a**,**b**) indicate the day of a year (DOY) and color bar in (**c**) indicates the length of a season in days.

In stage two of HCM, a feature space including three phenological parameters was constructed to distinguish the cropping frequency. According the unique phenological calendar of the crops, the first season of double crop starts on about DOY 033–049, ends their season on about DOY 113–129 and has a season length of about 80–96 days. However, the single crop starts their season on about DOY 145–161, ends their season on about DOY 257–273 and has a season length of about 96–112 days. These features can be observed in Figure 6a–c. So, SOS, EOS, LOS were combined to a 3-layers data and the IsoData unsupervised clustering method was used to get a finer cropping frequency map. In this clustering, maximum iterations were set to 20, expected number of classes was set to 4. Finally, two cropping frequency were obtained according to the clustering result: double crop (DC for short), single crop (SC for short) (Figure 5).

### 3.3. Spatial-Temporal Analysis of the Pattern

Hotspot analysis was used to express the spatial pattern of cropping frequency and Sen trend [30] test was used to measure the temporal pattern of cropping frequency. The temporal analysis was conducted at provincial level and county level respectively.

(1) Spatial Analysis

Hotspot analysis uses vectors to identify the locations of statistically significant hot spots and cold spots in data. Moran’s Index can indicate clustering or dispersion pattern in the data and *z* score and *p*-value evaluating the significance of that index. A high *z* score and small *p* value for a feature indicates a significant hot spot. A low negative *z* score and small *p* value indicates a significant cold spot. The higher (or lower) the *z* score, the more intense the clustering. A *z* score near zero means no spatial clustering.

Getis-Ord $G_{i}^{*}$ in ArcGIS 10.2 was used to calculate the Moran’s I Index value and both a *z* score and *p*-value. The Getis-Ord local statistic is given as:

(1) $$G_{i}^{*}=\frac{\sum_{j=1}^{n}w_{i,j}x_{j}-\bar{X}\sum_{j=1}^{n}w_{i,j}}{S\sqrt{\frac{n\sum_{j=1}^{n}w_{i,j}^{2}-{\left(\sum_{j=1}^{n}w_{i,j}\right)}^{2}}{n-1}}}$$

where $x_{j}$ is the attribute value for feature *j*, $w_{i,j}$ is the spatial weight between feature *i* and *j*, *n* is equal to the total number of features.

(2) Sen Trend Test

Sen method, a non-parametric trend test method proposed by Sen [30] was used to calculate the changing trend of crop area. The advantage of Sen trend analysis is that it does not require the sample to obey a certain distribution and is not influenced by outliers data or measurement error [31]. Its equation is as follows.

(2) $$\beta =\mathrm{mean}\left(\frac{x^{j}-x^{i}}{j-i}\right)\;\forall j>i$$

In this equation β is the trend of crop area, *i* and *j* indicate the interval of the time series, $x^{i}$ and $x^{j}$ indicate the EVI value for year *i* and *j*. If β is greater than zero, there is an increasing trend in the time-series and vice versa.

Mann-Kendall [32] test was performed to test the significance level of trend absence in the time-series data. The null hypothesis for this test is that there is no trend in the series. The alpha was set to 0.05. If the *p*-value of a test is less than alpha, the test rejects the null hypothesis. If the *p*-value is greater than alpha, there is insufficient evidence to reject the null hypothesis.

## 4. Results and Analysis

### 4.1. Validation of the Map

We collected cropland samples from time-series EVI data using Fishnet tools in ArcGIS at 0.25° interval and totally 625 samples were collected. We identified cropping frequency based on time-series EVI curve, combined with false color image and prior knowledge. The overall accuracy of sample validation based on visual identification was 88.5% (Kappa coefficient: 0.861) (Table 1). The slope of linear regression for double crop and single crop obtained from remotely sensed data and official statistical data was 0.84 (*R*^2^ = 0.79, *p* < 0.001) and 0.81 (*R*^2^ = 0.77, *p* < 0.001) respectively at county level, suggesting that this method is an effective way to map cropping frequency.

The above validation shows that the results of cropping frequency obtained by using the proposed method are reliable.

### 4.2. Spatial Pattern

**Figure 7:**
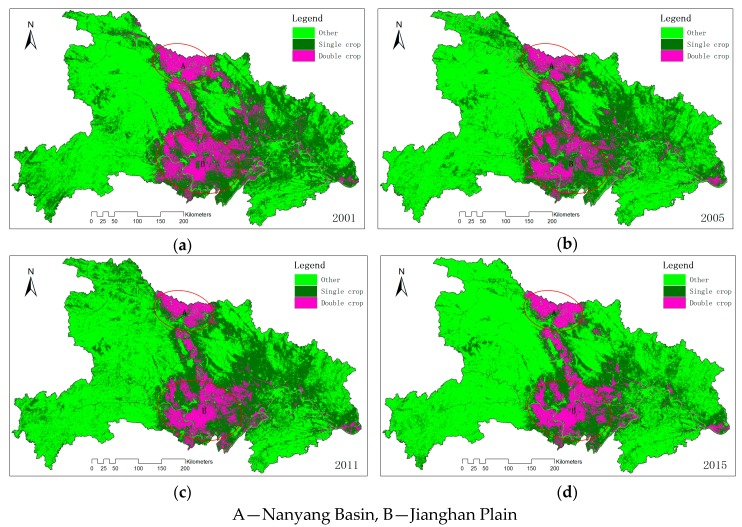
Cropping frequency maps, (**a**) 2001, (**b**) 2005, (**c**) 2011, (**d**) 2015.

The proposed HCM was applied to all the observed years to get the time-series cropping frequency maps. In these maps, natural vegetation, constructed surfaces, water bodies are all placed into one class: other. Figure 7 was the cropping frequency maps from 2001 to 2015 at five-year interval. We can observe that DC is mainly distributed on Nanyang Basin and Jianghan Plain, while SC is mainly distributed on central and eastern Hubei. DC and SC take up approximately 30% and 70% of the total croplands respectively in 2015.

**Figure 8:**
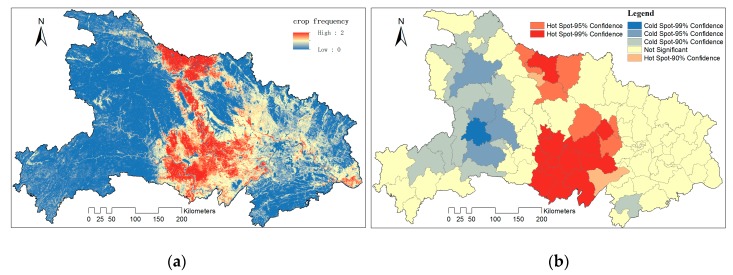
(**a**) The average cropping frequency of Hubei during 2001 and 2015; (**b**) Hot spot analysis of the average cropping frequency.

In cropping frequency maps, numbers 2, 1, 0 were used to indicate double crop, single crop and other respectively. We calculate the mean value of crop frequencies during 2001–2015 to get an average cropping frequency map of Hubei Province (Figure 8a). The areas with high frequency were found mainly on the core areas of Jianghan Plain, Hanjiang Basin and Nanyang Basin.

Local autocorrelation analysis of average cropping frequency indicates that the hot spot of DC includes most counties in Jianghan Plain, which was one of the main grains productive basements of China and all the counties within Nanyang Basin (Figure 8b).

### 4.3. Temporal Pattern

(1) The Change of Total Crop Areas

**Figure 9:**
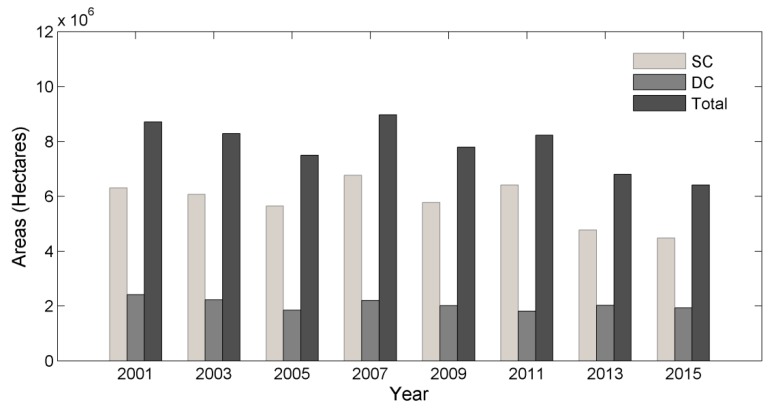
The crop areas of Hubei Province.

The crop areas for SC and DC were calculated using zonal statistics tool in ArcGIS at county level and then summarized to provincial level. We found that the total crop areas of Hubei Province dropped significantly, from 8,718,576 Ha in 2001 to 6,409,400 Ha in 2015. DC area decreased slightly, while SC area decreased significantly during these 15 years (Figure 9).

(2) The Change Trend at County Level

**Figure 10:**
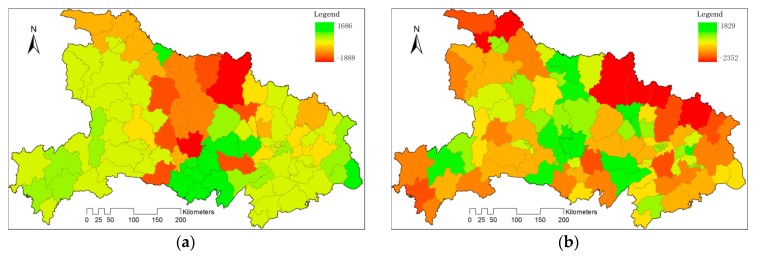
Sen trend maps of the cropping frequency at county level, (**a**) DC and (**b**) SC.

We explored the change trend of DC and SC at county level using Sen trend test. Figure 10 gives the Sen trend map of SC and DC at county level. Red and green indicate the crop area decrease or increase drastically. At county level, it exhibits distinctive characteristic for their trend. For DC, the counties with a significant decreasing trend mainly cover from Danjiangkou reservoir area to the north of Jianghan Plain. The counties with a significant increasing trend mainly cover the core area of Jianghan Plain. For SC, the counties with a significant decreasing trend mainly cover the marginal mountain areas, within which there are far less croplands. Within the main crop areas, the core areas of Jianghan Plain have a significant decreasing trend, while the areas from Nanyang Basin to the north of Jianghan Plain have a significant increasing trend.

**Figure 11:**
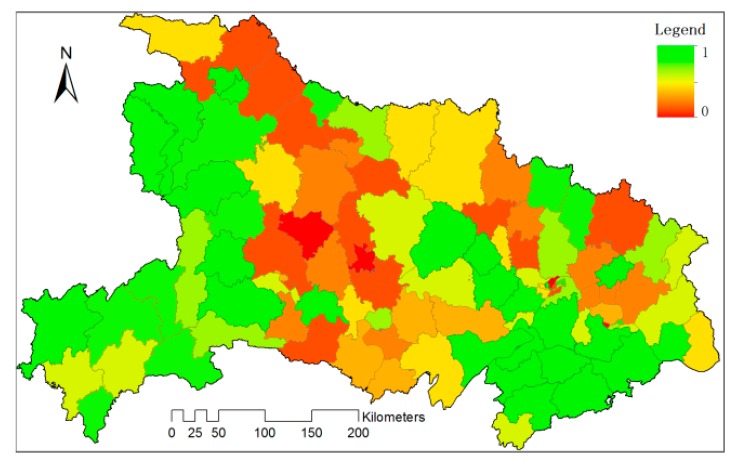
The significance level for the change of DC, at the confidence level of 0.05.

Sen trend maps of DC and SC were combined together to get a more meaningful pattern. In the core area of Jianghan Plain, the increasing of DC was along with the decreasing of SC. However, from Nanyang Basin to the north of Jianghang Plain, the decrease of DC occurred alongside the increase of SC. These changes indicate intensive transfer between DC and SC during these years. As for the Danjiangkou reservoir area, however, both the DC and SC have a trend of decrease, which indicates some abandon of the croplands in these areas. Furthermore, all these changes in DC are significant in above mentioned areas (Figure 11).

(3) The Transfer Between SC and DC

**Figure 12:**
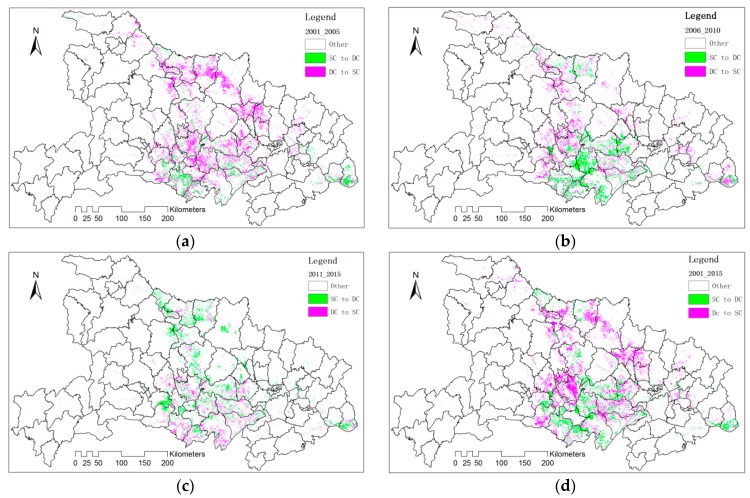
The transfer maps of SC and DC based on cropping frequency at five-year interval, (**a**) 2001–2005, (**b**) 2006–2010, (**c**) 2011–2015, (**d**) 2001–2015.

We selected those change pixels (SC transfer to DC or DC transfer to SC) to get a transfer map at 5-year interval (Figure 12). General speaking, the change was frequent during the observed years, covering almost most croplands. The decrease in cropping frequency can be observed at the north of the research area, including the north of Jianghan Plain. The changes of cropping frequency in Jianghan Plain were intensive and the transfer of SC to DC dominated the trend.

**Figure 13:**
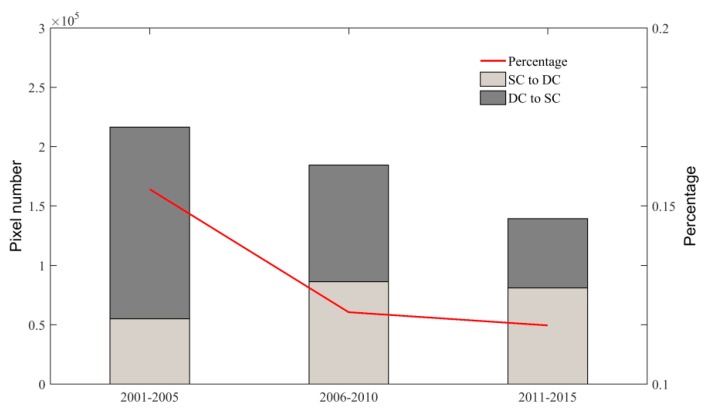
The amount and their percentage transferred between SC and DC.

We summarize the changed pixels (SC transfer to DC or DC transfer to SC) and their proportion to the total cropland pixels at 5-year interval (Figure 13). Overall, about 11~15% croplands changed their cropping frequency every 5 years. However, the total amount of the changes was slowing down since 2005. During 2001–2005, the amount DC transfer to SC was more than that of SC transfer to DC. However, this trend has been reversed during 2011–2015.

## 5. Discussion

The spatial-temporal dynamics of cropping frequency in Hubei Province lies in several aspects. Social and economic factors are primary reasons. The rapid urbanization and development of market economy impose their impact on agricultural production. It’s very often that the farmers abandon the fields and get a work in cities, or aquaculture for aquatic products, to obtain a higher income. The change in crop patterns also has some relationships with the shortage in water resources caused by man-made huge constructions. The decrease of DC can be mostly observed in areas from Danjiangkou reservoir area to the north of Jianghan Plain (Figure 10a). All these counties have a significance level lower than 0.05 (Figure 11). Chinese government initialed a water project, South-to-North Water Transfer Project in 2003. Danjiangkou reservoir area is the water resource of The Central Route of South-to-North Water Transfer Project. The shortage in water resources influenced the irrigation of croplands in the Hanjiang River Valley, then the crop pattern.

We would highlight the findings that there is obvious regional differentiation of temporal patterns of cropping frequency in Hubei Province, showing diversified trends in different regions (see Section 4.3). From Danjiangkou reservoir area to Nanyang Basin and the Northern margin of Jianghan Plain, water shortage is the main reason. However, non-traditional agricultural areas are more affected by the rapid development of market economy. As for Jianghan Plain, it’s more influenced by the national policy and the crop patterns keep changing. Therefore, we propose some suggestions for the agricultural planning of Hubei Province. Firstly, we recommend discriminating crop patterns in different regions of Hubei Province. To be specific, North Hubei should give priority to the development of SC (mainly rain-fed crops), minimizing the dependence on water. Jianghan Plain should attach great importance to the development of DC and maintain the dominance of Jianghan Plain in Hubei’s agricultural production. Secondly, the government should encourage farmer’s willingness to plant by policy regulation. The farmers should expand the DC areas by reducing the abandonment of croplands. Through these efforts, we can bridge the yield gap by making full use of the potential of agricultural production.

The accuracy of the cropping frequency maps inevitably has some uncertainties, partly because the mixed pixels caused by coarse resolution of the MODIS data and the heterogeneity of the land covers. There is still much noise and fluctuation in the time-series EVI data, resulting in uncertainty when extracting the phenological parameters.

## 6. Conclusions

Spatial-temporal patterns of cropping frequency provide relevant information for management and decision-making policies. This paper reports our work on exploring the spatial-temporal patterns of crop system in Hubei Province of mainland China from time-series MODIS data. A hierarchical clustering method was proposed to map cropping frequency in two successive stages and the spatial and temporal patterns of cropping frequency from 2001 to 2015 in Hubei Province were analyzed. We found that essential changes on crop patterns have taken place in Hubei Province during 2001–2015 and these changes have distinct regional differentiation. Overall, the total DC areas decreased slightly, while SC areas decreased significantly. The transfer between DC and SC was frequent with approximately 11~15% croplands changing their cropping frequency every 5 years. The significant changes at county level include: the increase of DC along with the decrease of SC in the core area of Jianghan Plain, the decrease of DC along with the increase of SC from Nanyang Basin to the north of Jianghang Plain and a trend of decrease on both DC and SC on the Danjiangkou reservoir area.

The contributions of our work are summarized as follows. To begin with, the proposed method has the superiority of mapping cropping frequency accurately at regional scale at long time-series. HCM combined time-series EVI data and phenological parameters therefore can be a good solution to get a more accurate cropping frequency map. Secondly, we found that the proposed method can reveal the dynamics of crop patterns very well. Sen method and Mann-Kendall test together can figure out the trend of the changes and also the significance level of trend existing in the time-series data. In addition, spatial and temporal patterns of cropping frequency in Hubei Province were analyzed from the aspect of the total change, the transfer between DC and SC and the regional differentiation of the change at county level. Finally, we recommended discriminating crop patterns in different regions of Hubei Province according to the regional differentiation of spatial-temporal dynamics of cropping frequency.

Future work will involve the analysis of spatial and temporal variations of the crop phenology, which have important implications for predicting the potential impacts of climate change on food security in the future.

## Figures and Tables

**Table 1:** Confusion matrix of cropping frequency map.

Extracted by the Algorithm	Artificial Interpretation				
	Single Crop	Double Crop	Other	Total	User’s Accuracy (%)
Single crop	159	6	12	177	89.8
Double crop	8	73	6	96	83.4
other	21	5	310	336	92.3
total	188	84	328	625	--
Producer’s accuracy (%)	84.5	86.9	87.8	--	--

## References

[1] Schmidhuber J., Tubiello F.N. (2007). Global food security under climate change. Proc. Natl. Acad. Sci. USA.

[2] Peña-López I. (2008). Global Economic Prospects 2008: Technology Diffusion in the Developing World.

[3] Frolking S., Xiao X., Zhuang Y., Salas W., Li C. (1999). Agricultural land-use in China: A comparison of area estimates from ground-based census and satellite-borne remote sensing. Glob. Ecol. Biogeogr..

[4] Hurtt G.C., Rosentrater L., Frolking S., Moore B. (2001). Linking remote-sensing estimates of land cover and census statistics on land use to produce maps of land use of the conterminous United States. Glob. Biogeochem. Cycles.

[5] Yan H., Xiao X., Huang H., Liu J., Chen J., Bai X. (2014). Multiple cropping intensity in China derived from agro-meteorological observations and MODIS data. Chin. Geogr. Sci..

[6] Tang H., Wu W., Yang P., Zhou Q., Chen Z. (2010). Recent progresses in monitoring crop spatial patterns by using remote sensing technologies. Sci. Agric. Sin..

[7] Esquerdo J., Zullo Júnior J., Antunes J. (2011). Use of NDVI/AVHRR time-series profiles for soybean crop monitoring in Brazil. Int. J. Remote Sens..

[8] Arvor D., Jonathan M., Meirelles M.S.P., Dubreuil V., Durieux L. (2011). Classification of MODIS EVI time series for crop mapping in the state of Mato Grosso, Brazil. Int. J. Remote Sens..

[9] Brown J.C., Kastens J.H., Coutinho A.C., de Castro Victoria D., Bishop C.R. (2013). Classifying multiyear agricultural land use data from Mato Grosso using time-series MODIS vegetation index data. Remote Sens. Environ..

[10] Victoria D.d.C., da Paz A.R., Coutinho A.C., Kastens J., Brown J.C. (2012). Cropland area estimates using Modis NDVI time series in the state of Mato Grosso, Brazil. Pesqui. Agropecu. Bras..

[11] Lv T., Liu C. (2010). Study on extraction of crop information using time-series MODIS data in the Chao Phraya Basin of Thailand. Adv. Space Res..

[12] Chen C.F., Son N., Chang L., Chen C. (2011). Classification of rice cropping systems by empirical mode decomposition and linear mixture model for time-series MODIS 250 m NDVI data in the Mekong Delta, Vietnam. Int. J. Remote Sens..

[13] Chen C., Son N., Chang L. (2012). Monitoring of rice cropping intensity in the upper Mekong Delta, Vietnam using time-series MODIS data. Adv. Space Res..

[14] Atzberger C., Rembold F. (2013). Mapping the spatial distribution of winter crops at sub-pixel level using AVHRR NDVI time series and neural nets. Remote Sens..

[15] Mkhabela M., Bullock P., Raj S., Wang S., Yang Y. (2011). Crop yield forecasting on the Canadian Prairies using MODIS NDVI data. Agric. For. Meteorol..

[16] Wardlow B.D., Egbert S.L., Kastens J.H. (2007). Analysis of time-series MODIS 250 m vegetation index data for crop classification in the US Central Great Plains. Remote Sens. Environ..

[17] Wardlow B.D., Egbert S.L. (2010). A comparison of MODIS 250-m EVI and NDVI data for crop mapping: A case study for southwest Kansas. Int. J. Remote Sens..

[18] Lunetta R.S., Shao Y., Ediriwickrema J., Lyon J.G. (2010). Monitoring agricultural cropping patterns across the Laurentian Great Lakes Basin using MODIS-NDVI data. Int. J. Appl. Earth Obs. Geoinf..

[19] Conrad C., Colditz R.R., Dech S., Klein D., Vlek P.L. (2011). Temporal segmentation of MODIS time series for improving crop classification in Central Asian irrigation systems. Int. J. Remote Sens..

[20] Vintrou E., Desbrosse A., Bégué A., Traoré S., Baron C., Seen D.L. (2012). Crop area mapping in West Africa using landscape stratification of MODIS time series and comparison with existing global land products. Int. J. Appl. Earth Obs. Geoinf..

[21] Vrieling A., de Beurs K.M., Brown M.E. (2011). Variability of African farming systems from phenological analysis of NDVI time series. Clim. Chang..

[22] Estel S., Kuemmerle T., Alcántara C., Levers C., Prishchepov A., Hostert P. (2015). Mapping farmland abandonment and recultivation across Europe using MODIS NDVI time series. Remote Sens. Environ..

[23] Pan Y., Li L., Zhang J., Liang S., Zhu X., Sulla-Menashe D. (2012). Winter wheat area estimation from MODIS-EVI time series data using the Crop Proportion Phenology Index. Remote Sens. Environ..

[24] Pan Z., Huang J., Zhou Q., Wang L., Cheng Y., Zhang H., Blackburn G.A., Yan J., Liu J. (2015). Mapping crop phenology using NDVI time-series derived from HJ-1 A/B data. Int. J. Appl. Earth Obs. Geoinf..

[25] Ding M., Chen Q., Xin L., Li L., Li X. (2015). Spatial and temporal variations of multiple cropping index in China based on SPOT-NDVI during 1999–2013. Acta Geogr. Sin..

[26] Huang Q., Tang H., Zhou Q., Wu W., Wang L., Zhang L. (2010). Remote-sensing based monitoring of planting structure and growth condition of major crops in Northeast China. Trans. Chin. Soc. Agric. Eng..

[27] Huete A., Didan K., Miura T., Rodriguez E.P., Gao X., Ferreira L.G. (2002). Overview of the radiometric and biophysical performance of the MODIS vegetation indices. Remote Sens. Environ..

[28] Jönsson P., Eklundh L. (2004). TIMESAT—A program for analyzing time-series of satellite sensor data. Comput. Geosci..

[29] Jönsson P., Eklundh L. (2010). TIMESAT 3.0 Software Manual.

[30] Sen P.K. (1968). Estimates of the regression coefficient based on Kendall’s tau. J. Am. Stat. Assoc..

[31] Liu X., Pan Y., Zhu X., Li S. (2015). Spatiotemporal variation of vegetation coverage in Qinling-Daba Mountains in relation to environmental factors. Acta Geogr. Sin..

[32] Kendall M.G. (1948). Rank Correlation Methods.

